# A massive parallel sequencing workflow for diagnostic genetic testing of mismatch repair genes

**DOI:** 10.1002/mgg3.62

**Published:** 2014-01-21

**Authors:** Maren F Hansen, Ulrike Neckmann, Liss A S Lavik, Trine Vold, Bodil Gilde, Ragnhild K Toft, Wenche Sjursen

**Affiliations:** 1 Department of Laboratory Medicine, Children and Women's Health, Faculty of Medicine, Norwegian University of Science and TechnologyTrondheim, Norway; 2 Department of Pathology and Medical Genetics, St. Olavs HospitalTrondheim, Norway

**Keywords:** Amplicon sequencing, diagnostics, hereditary colorectal cancer, massive parallel sequencing, mismatch repair, *MLH1*, *MSH2*, *MSH6*, *PMS2*

## Abstract

The purpose of this study was to develop a massive parallel sequencing (MPS) workflow for diagnostic analysis of mismatch repair (MMR) genes using the GS Junior system (Roche). A pathogenic variant in one of four MMR genes, (*MLH1, PMS2, MSH6*, and *MSH2*), is the cause of Lynch Syndrome (LS), which mainly predispose to colorectal cancer. We used an amplicon-based sequencing method allowing specific and preferential amplification of the MMR genes including *PMS2*, of which several pseudogenes exist. The amplicons were pooled at different ratios to obtain coverage uniformity and maximize the throughput of a single-GS Junior run. In total, 60 previously identified and distinct variants (substitutions and indels), were sequenced by MPS and successfully detected. The heterozygote detection range was from 19% to 63% and dependent on sequence context and coverage. We were able to distinguish between false-positive and true-positive calls in homopolymeric regions by cross-sample comparison and evaluation of flow signal distributions. In addition, we filtered variants according to a predefined status, which facilitated variant annotation. Our study shows that implementation of MPS in routine diagnostics of LS can accelerate sample throughput and reduce costs without compromising sensitivity, compared to Sanger sequencing.

## Introduction

Sanger sequencing (Sanger et al. 1977) has been the gold standard for DNA sequencing during the past decades. However, since the commercial launch of the first massive parallel sequencing (MPS) platform, the Genome Sequencer FLX System from 454 Life Sciences (Roche) in 2005, the sequencing technology has undergone a rapid development (Margulies et al. 2005). MPS has especially been embraced by genomic research because it facilitates performance of complex genetic studies that were not technically or economically feasible with Sanger sequencing. Use of MPS in genetic diagnostics was limited in its initial phase, mostly due to the costs and capacity of the first MPS platforms. Genome and exome sequencing are examples of applications that these platforms are designed for, while for clinical applications sequencing of subsets of genes are of most interest. In 2010, Roche introduced the GS Junior, which is a small benchtop sequencing platform more compatible with the needs of a diagnostic laboratory. This system produces 100,000 shotgun and 70,000 amplicon high-quality, filtered reads, each 10-h run. Average read length is 400 bp. The performance of this system should be comparable to the well-documented GS FLX System (Rothberg and Leamon 2008; Liu et al. 2012) because they both use 454 sequencing chemistry, which combines emulsion polymerase chain reaction (PCR) of single-stranded DNA molecules and massive parallel pyrosequencing.

Usually, the DNA variant detection strategy in a diagnostic laboratory consists of a screening method like high-resolution melting (HRM) analysis followed by confirmation of the detected variant by Sanger sequencing. The large capacity of MPS platforms and the possibility to multiplex samples can improve screening efficiency. Several papers have presented MPS protocols for diagnostic purposes, the vast majority for analysis of *BRCA1* and *BRCA2* (Morgan et al. 2010; Walsh et al. 2010; De Leeneer et al. 2011b; Feliubadalo et al. 2012; Hernan et al. 2012; Michils et al. 2012) but also Neurofibromatosis type 1 (Chou et al. 2010). Different strategies have been utilized, like sequence capture with subsequent MPS (Chou et al. 2010; Walsh et al. 2010) and commercial or in-house amplicon-based sequencing methods (Morgan et al. 2010; De Leeneer et al. 2011b; Feliubadalo et al. 2012; Hernan et al. 2012; Michils et al. 2012). For this study, we selected the DNA mismatch repair (MMR) genes *MSH2* (MIM #609309), *MLH1* (MIM #120436), *MSH6* (MIM #600678), and *PMS2* (MIM #600259) to optimize a Massive parallel amplicon-sequencing analysis. Mutations in any of these genes are the causes of most Lynch Syndrome (LS) cases (Peltomaki 2005). LS, also known as Hereditary Nonpolyposis Colorectal Cancer (HNPCC), predispose to colorectal cancer (CRC) and are autosomal dominantly inherited. It is the most common hereditary CRC syndrome and account for 3–4% of all CRCs (Hampel et al. 2008). Extracolonic cancers in the endometrium, ovary, stomach, hepatobiliary tract, upper urinary tract, small bowel, pancreas, and brain are also associated with LS (reviewed in Bozzao et al. 2011).

*PMS2* analysis is often neglected from genetic testing of LS due to the presence of multiple pseudogenes. Strong homology between several pseudogenes and gene sequence introduce difficulties for reliable variant detection (Nicolaides et al. 1995; De Vos et al. 2004; Nakagawa et al. 2004). There are 15 different *PMS2* pseudogene loci identified in the human genome. The majority share homology with the 5′ end of *PMS2* containing exon 1–5 while the pseudogene *PMS2CL* share homology with exons 9 and 11–15, where exon 12 and 15 are identical to *PMS2* (De Vos et al. 2004; Nakagawa et al. 2004). In addition, sequence exchange between the 3′ region of *PMS2* and the pseudogene *PMS2CL* may cause detection of pseudogene sequence (Hayward et al. 2007). To our knowledge, we are the first to present a MPS workflow for diagnostic analysis of the MMR genes associated with LS, including *PMS2*. Careful primer design that utilizes differences in the *PMS2*-gene and pseudogenes was essential to avoid coamplification and subsequent sequencing of pseudogenes.

We optimized an in-house amplicon-sequencing approach analyzed on the GS Junior system. This study provides an example of how this can be performed starting with amplicons originally designed for Sanger sequencing and using standard data analysis software, accompanying the sequencing system. The strategies for achieving coverage uniformity and dealing with under or overcalling of homopolymer (HP) stretches are different from previously reported. The MPS workflow is compared to Sanger sequencing with regard to, workload, sample turnaround time, specificity, and sensitivity.

## Materials and Methods

### DNA samples

In total, 55 DNA samples isolated from ethylenediaminetetraacetic acid preserved whole blood with iPrep Pure link gDNA Blood kit were included in the study. All patient samples were obtained with written informed consent.

The capability of the GS Junior system to detect insertion and deletion variants was evaluated using 23 samples previously characterized by Sanger sequencing in a diagnostic setting. The previously identified variants were 14 deletions, seven duplications, and two indels. These samples were sequenced only for the amplicon containing the variant (coverage results not shown).

To setup and optimize our MMR gene MPS workflow, 32 samples included in a local CRC biobank described elsewhere (Trano et al. 2010) were sequenced for *MLH1, PMS2*,*MSH6,* and *MSH2*. For 16 of these samples, one or several MMR genes had previously been Sanger sequenced (six samples for all four MMR genes, one sample for *MLH1, MSH2,* and *PMS2*, three samples for *MLH1* and *PMS2*, three samples for *MSH2* and *MSH6* and one sample was only sequenced for *PMS2*). Only the previously identified variants were used for evaluation of MPS sensitivity. The remaining 16 samples, previously uncharacterized, were used to test the workflow in a diagnostic setting.

### Library preparation

**Figure 1:**
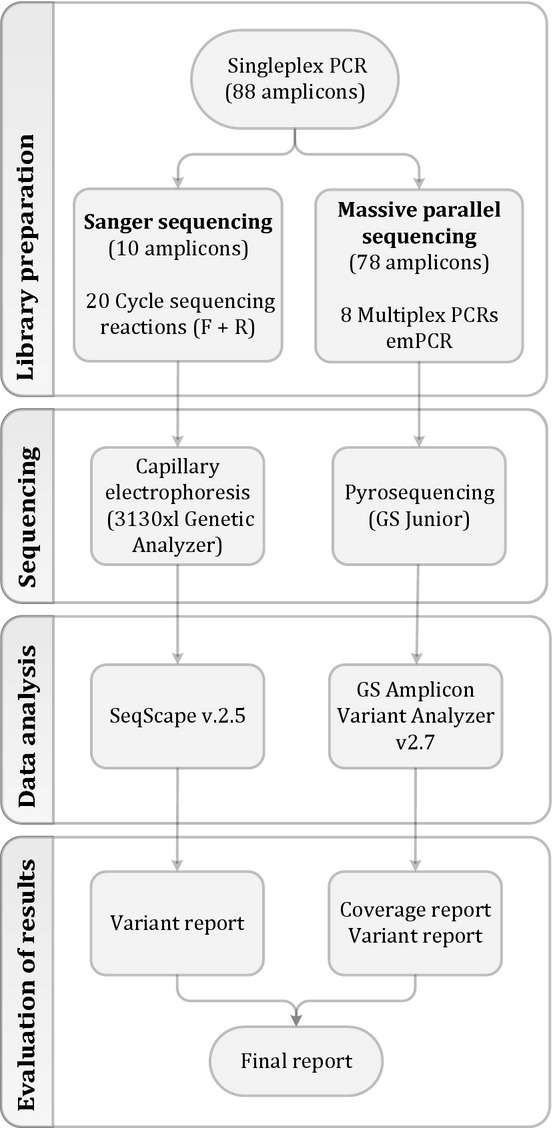
Workflow for sequencing the MMR genes. For each sample 88 singleplex, PCRs are setup. The workflow is then separated into two simultaneous workflows. 10 amplicons unsuited for MPS are analyzed by Sanger sequencing. This generates 20 cycle sequencing reactions (forward and reverse direction) that are analyzed with capillary electrophoresis using a 3130xl Genetic Analyzer. Data analysis is done with the software SeqScape v. 2.5 and a variant report is generated. The remaining 78 amplicons are pooled into eight multiplex PCRs that adds the sample-specific MIDs. Subsequently, all multiplex reactions to be analyzed in a single-GS Junior run are pooled to a total pool prior to the emulsion PCR. Sequencing of the enriched amplicons is performed by the GS Junior benchtop sequencer from Roche. Data analysis is done with the GS Amplicon Variant Analyzer (AVA) software and a variant report is generated. Ultimately, variant reports are combined to a final result report for each patient and test results are sent to the requisitioner.

We used an in-house amplicon-sequencing procedure similar to De Leeneer et al. (2011b). Only minor modifications to our original Sanger amplicon design were necessary to make it suitable for MPS. The workflow consisted of two parallel parts, one MPS part and one Sanger sequencing part (Fig. 1). The MPS procedure involved two rounds of PCR. First, a target-specific singleplex PCR, using fusion primers with universal tail sequences. Amplicons from the singleplex PCRs were pooled before the second multiplex PCR was carried out, using MID (Multiplex Identifier)-barcoded primers targeting the universal tails in the first PCR round. MID-barcodes are used by the GS Junior system software to identify the individual patient samples. All PCRs were performed on the Techne TC-512 thermal cycler.

#### Singleplex universal-tailed PCR

For each sample, 88 fragments were amplified to cover the complete coding and splice site regions of *MSH2, MSH6, MLH1*, and *PMS2*. The amplicon lengths ranged from 283 to 489 bp. PCR setup was performed by Hamilton STARlet liquid handling workstation. The total volume for this PCR was 25 *μ*L and included 2× SensiMix™ HRM (Bioline, London, U.K.), 1.4 mmol/L MgCl_2_, 0.28 *μ*mol/L of each primer (forward and reverse), and 30 ng DNA. Three different touchdown PCR programs were used. The thermal cycling conditions were: denaturation at 95°C for 10 min, then 16/8 cycles of denaturation at 95°C for 30 sec, annealing at 61/64/66°C (decreasing 0.5°C for each cycle), and extension at 72°C. Then, 24/32 additional cycles followed consisting of denaturation at 95°C for 30 sec, annealing at 53/56/62°C for 30 sec, and extension at 72°C. Final extension was carried out at 72°C for 5 min.

#### MID-barcoded multiplex PCR

After the first PCRs (singleplex universal-tailed PCR), 78 (10 were Sanger sequenced) singleplex amplicons were pooled to eight amplicon pools for each sample. To ensure uniform coverage distribution, the different singleplex amplicons were pooled at different ratios with volumes varying from 1 *μ*L to 20 *μ*L depending on the coverage obtained in the previous sequencing analysis. Composition of the different amplicon pools are shown in Table A1. Each pool was diluted 100 times and 1 *μ*L was used as template for the multiplex PCR with MID-barcoded primers. The amplification mixture included 5× AccuPrime™GC-Rich Buffer A (Life Technologies, Paisley, U.K.), 1 U Accuprime™ GC-Rich DNA Polymerase (Life Technologies), and 0.28 *μ*mol/L of each MID-barcoded primer (forward and reverse). Total volume for this PCR was 25 *μ*L. The PCR program consisted of a 5 min denaturation step at 95°C, 20 cycles of denaturation at 95°C for 30 sec, annealing at 58°C for 30 sec, extension at 72°C for 1 min, and final extension at 72°C for 5 min.

The eight multiplex PCR products were pooled to one total sample pool that consisted of all 78 amplicons from one sample. The pools were purified with Agencourt® AMPure® XP (Beckman Coulter, High Wycombe, U.K.) according to the library purification procedure described by Roche in the Amplicon Library Preparation Method Manual. Bioanalyzer 2100 (Agilent, Santa Clara, CA) was used to evaluate the fragment lengths in the amplicon pools. DNA concentration of the pools was measured with Nanodrop 8000 Spectrophotometer (Thermo Scientific, Wilmington, DE) and the eight total sample pools were equimolarly pooled again into one mix containing all amplicons from all samples to be sequenced in a single-GS Junior run.

### Massive parallel sequencing

The emulsion PCR protocol (Lib-A) recommends an input of two molecules of library DNA per capture bead. Based on our own (unpubl. data) and others (Zheng et al. 2010; Jiang et al. 2012) experimental experience, a lower molecule-to-bead ratio gives more desirable sequencing results because the amount of nonreadable beads with more than one DNA molecule is reduced. We used a molecule per bead ratio of 0.5 and obtained an enrichment percentage of ∼5% (500,000 beads containing DNA). Loading only 500,000 beads (as opposed to 2,000,000 beads, which is the upper limit) onto the Pico Titer Plate (PTP), reduce light signal interference from neighboring wells and further increase sequencing quality. Sequencing on the GS Junior was performed according to manufacturer's instructions.

### Data analysis

The reads from each GS Junior run were analyzed using GS Amplicon Variant Analyzer v2.7 (AVA) software (Roche, Basel, Switzerland). All reads that passed the AVA default filters were aligned to *PMS2* (NG_008466.1), *MSH6* (NG_007111.1), *MLH1* (NG_007109.1), and *MSH2* (NG_007110.1). We applied some additional filters, which we considered useful to reduce the number of false positives (FP) without risk of losing any true positives (TP). Only variants present in both forward and reverse reads, with a combined variant frequency (VF) of at least 15% were further considered. Theoretically, this filter setting requires a minimum coverage of 18 to detect a heterozygote variant with a probability of 99.9% (Phred score of 30) (De Leeneer et al. 2011a). However, as this theoretical value only takes sampling effects into consideration, we found it to be too low. Our experience (see below) is that sequence context also affects allele frequencies and we therefore elevated the coverage threshold to 38. This threshold has also been used in other studies (De Leeneer et al. 2011b; Feliubadalo et al. 2012).

**Figure 2:**
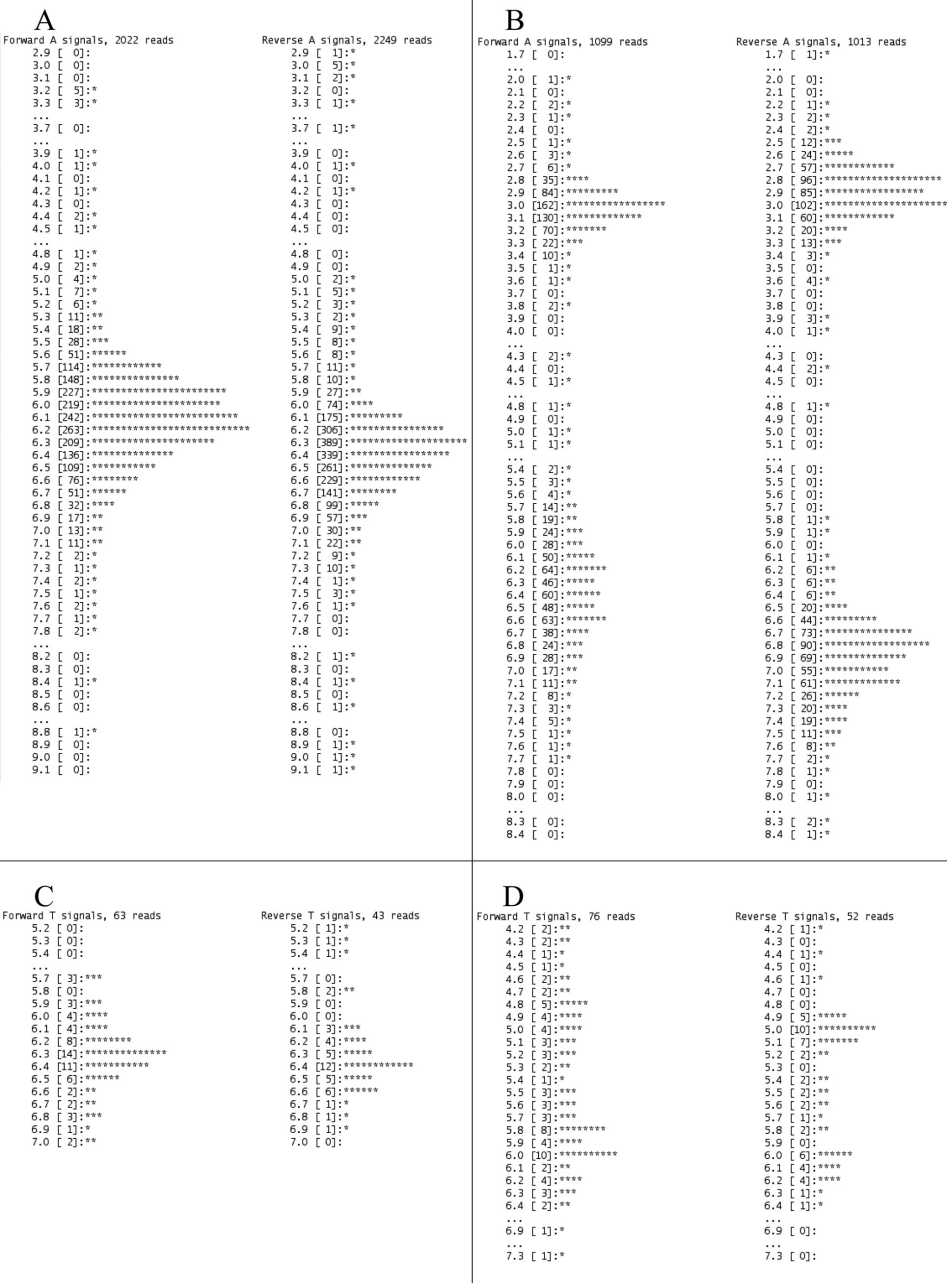
Histograms of signal distributions from FP and TP variants. (A) Distribution from an overcall of a homopolymeric stretch of 6-mer As. The stretch was called containing 7-mer As in 23% of the reads. A single peak around 6-mer with long tails indicates a false-positive call. (B) Signal distribution from a true call (c.680_683del in *MSH2*) leading to the sequence change GAAA*GAAA*AAAAG→GAAAAAAAG. This distribution shows strong evidence for both 3-mer and 7-mer. Dual peaks with distributions for both forward and reverse reads centered on the values 3-mer and 7-mer indicate a true-positive variant. (C) Signal distribution from an overcall of a homopolymeric stretch of 6-mer Ts. The stretch was called containing 7-mer Ts in 24% of the reads. A single peak around 6-mer Ts with long tails indicates a false-positive call. (D) Signal distribution from a true-positive call (c.*85T>A in *MSH6*) of a variant located between two homopolymeric regions (TTTTT*T*AAAAA). This distribution indicates both 5-mer and 6-mer Ts for this homopolymeric region. Note that histogram (A and B) are made up of more reads than histogram (C and D). Higher coverage gives nicer distributions and facilitate interpretation.

Under and overcalling of HP regions is a well-known problem with pyrosequencing (Huse et al. 2007). To separate TP from FP calls in HP regions, we did a cross-sample comparison and evaluation of flow signal distributions (approach recommended by Roche). If the variant was present at similar frequency in forward and reverse directions across all samples, the variant was considered to be a FP. A signal distribution is a histogram of all the flow signals of forward and reverse reads that align to a specific position. When viewing flow signal distributions, the TP variants are expected to give dual peaks, while single peaks are expected in case of FP variants (Fig. 2). Note that evaluation of distribution signals requires that the variant is called with sufficient reads in the forward and reverse direction. Identified TP variants were annotated according to Human Genome Variation Society guidelines using transcript references NM_000251.1 (*MSH2*), NM_000249.3 (*MLH1*) NM_000179.2 (*MSH6*), and NM_000535.5 (*PMS2*), and classified using a five class system (1 = neutral, 2 = likely neutral, 3 = uncertain, 4 = likely pathogenic, and 5 = pathogenic) (Plon et al. 2008; Spurdle 2010). We further utilized the “variant status” filter in AVA that allows users to filter the variants based on their predetermined status. Variants can be set as either “accepted,” “putative,” or “rejected,” We found it useful to define all recurrent HP FP variants as rejected and all polymorphic variants (nonpathogenic) as accepted. All remaining variants (nonpolymorphic and new FPs) identified was set as putative by the software. In this way, we could easily recognize and distinguish FPs and common nonpathogenic variants from potentially disease causing variants.

The AVA software includes a Command Line Interface (CLI) that was utilized to get coverage reports on each run, as the graphical user interface (GUI) does not provide this function. The script for retrieving coverage report is available by request.

### Sanger sequencing

For each patient, 10 amplicons were Sanger sequenced after singleplex PCR. Of these, five amplicons contained HP regions ranging from 13 to 26 repeats and thereby caused base calling problems. Because of high similarity between *PMS2* and *PMS2CL,* the primer pairs amplifying exons 13–15 had to be located in deep intron sequences to find sequences differences. Consequently, the corresponding amplicons were too long (575, 738, and 771 bp) to be sequenced by this MPS approach. The remaining two amplicons were Sanger sequenced because during previous runs they were consistently undercovered (<38 reads), even when including the entire singleplex volume in the multipliex. In addition, all nonpolymorphic variants, putative variants that could not be confidently determined as TP or FP and undercovered amplicons (<38-fold coverage) in the MPS workflow were also Sanger sequenced. Cycle sequencing reaction was performed with BigDye® Terminator v3.1 (Life Technologies) and subsequent capillary electrophoresis was performed by the 3130xl Genetic Analyzer (Life Technologies). Sanger data were analyzed using SeqScape Software v2.5 (Life Technologies).

### cDNA sequencing to confirm PMS2 variants

To confirm that identified *PMS2* variants truly originate from the *PMS2* gene rather than any of the pseudogenes, we performed cDNA sequencing on seven samples containing *PMS2* variants identified in exons or exon/intron boundaries. RNA was isolated according to manufacturer's description from PAXgene Blood RNA tubes (Qiagen, Venlo, Limburg, Netherlands) using the nucleic acid purification kit PAXgene Blood RNA kit (Qiagen). We performed one-step RT-PCR to amplify the entire *PMS2* transcript in two overlapping fragments. Exon 10 was utilized as an anchor for reverse primer for the first fragment spanning exons 1–10, and forward primer for the second fragment spanning exons 10–15. As exon 10 is not present in any of the pseudogenes, this design ensures a specific amplification of PMS2. For One-step RT-PCR, <200 ng RNA was amplified in 25 *μ*L reactions using 0.2 *μ*g of each primer, the enzyme mix SuperScript®III One–Step RT-PCR Platinum® Taq HiFi (Life Technologies), and 2× Reaction Mix containing 0.4 mmol/L of each dNTP. Cycling consisted of an initial cDNA synthesis step at 55°C in 30 min followed by denaturation at 94°C for 2 min. For the first fragment, denaturation step was followed by 38 cycles of 15 sec at 94°C, 30 sec at 59°C, and 2.5 min at 68°C. For the second fragment, denaturation was followed by 40 cycles of 15 sec at 94°C, 30 sec at 55°C, and 3.25 min at 68°C. Final extension was carried out at 68°C in 7 min for both fragments. The RT-PCR products were sequenced (as explained above) using primers amplifying the regions spanning the variants that were to be confirmed.

## Results

Initially, 23 samples were sequenced only for the amplicons containing previously identified indel variants. Subsequently, we sequenced the four MMR genes *MSH2*,*MLH1*,*MSH6*, and *PMS2* in 32 patients divided on four GS Junior runs. Of these, 16 samples (run 1 and 2) were previously characterized with Sanger sequencing and were, together with the initial 23 samples containing indel variants, used to assess sensitivity and specificity of the GS Junior platform. The remaining 16 samples (run 3 and 4) were sequenced to test the utility of MPS workflow in a routine diagnostic setting. All variants identified in this study are presented in Table 1. From run 1 to 4, a strong optimization was performed to achieve uniform coverage distribution across all amplicons by adjusting amplicon pool ratios.

**Table 1:** All true-positive variants detected by massive parallel sequencing.

Gene^1^	DNA	dbSNP rsID	Protein	Class^2^	# Samples
*MLH1*	c.-7C>T	rs104894994	p.(=)	3	1
*MLH1*	c.-28A>G	rs56198082	p.(=)	3	1
*MLH1*	c.-93G>A	rs1800734	p.(=)	1	10
*MLH1*	c.39_40dup^3^	Not found	p.(Thr14Argfs^*^4)	5	1
*MLH1*	c.655A>G	rs1799977	p.(Ile219Val)	1	19
*MLH1*	c.866_867del^3^	Not found	p.(His289Profs^*^17)	5	1
*MLH1*	c.1411_1414del^3^	rs63751592	p.(Lys471Aspfs^*^19)	5	1
*MLH1*	c.1558+14G>A	rs41562513	p.(=)	1	2
*MLH1*	c.1668-19A>G	rs9876116	p.(=)	1	22
*MLH1*	c.1771dup^3^	Not found	p.(Asp591Glyfs^*^2)	5	1
*MLH1*	c.1852_1853delinsGC^3^	rs35502531	p.(Lys618Ala)	1	1
*MLH1*	c.1959G>T	rs1800146	p.(=)	2	2
*MLH1*	c.^*^35_^*^37del^3^	rs193922366	p.(=)	2	3
*MSH2*	c.-118T>C	rs2303425	p.(=)	1	9
*MSH2*	c.211+9C>G	rs2303426	p.(=)	1	25
*MSH2*	c.571_573del^3^	Not found	p.(Leu191del)	4	1
*MSH2*	c.680_683del^3^	Not found	p.(Arg227Lysfs^*^18)	5	1
*MSH2*	c.965G>A	rs4987188	p.(Gly322Asp)	2	2
*MSH2*	c.969_970del^3^	Not found	p.(Gtn324Valfs^*^8)	5	1
*MSH2*	c.1511-9A>T	rs12998837	p.(=)	1	9
*MSH2*	c.1661+12G>A	rs3732183	p.(=)	1	21
*MSH2*	c.1666T>C	rs61756466	p.(=)	3	1
*MSH2*	c.1705_1706del^3^	rs63751463	p.(Glu569Ilefs^*^2)	5	1
*MSH2*	c.1759G>C	rs63751140	p.(Gly587Arg)	5	1
*MSH2*	c.1786_1788del	rs63749831	p.(Asn596del)	4	1
*MSH2*	c.2006-6T>C	rs2303428	p.(=)	1	8
*MSH2*	c.2120_2122delins14^3^	Not found	p.(Cys707Serfs^*^3)	5	1
*MSH6*	c.-159C>T	rs41540312	p.(=)	1	11
*MSH6*	c.116G>A	rs1042821	p.(Gly39Glu)	1	3
*MSH6*	c.186C>A	rs1042820	p.(=)	1	11
*MSH6*	c.260+22C>G	rs55927047	p.(=)	1	11
*MSH6*	c.276A>G	rs1800932	p.(=)	1	10
*MSH6*	c.540T>C	rs1800935	p.(=)	1	19
*MSH6*	c.628-56C>T	rs1800936	p.(=)	1	11
*MSH6*	c.642C>T	rs1800937	p.(=)	1	12
*MSH6*	c.1186C>G	rs2020908	p.(Leu396Val)	2	3
*MSH6*	c.1405del^3^	Not found	p.(Tyr469Ilefs^*^12)	5	1
*MSH6*	c.1943del^3^	Not found	p.(Ser648Metfs^*^6)	5	1
*MSH6*	c.2302_2304del^3^	rs63750647	p.(Pro768del)	5	1
*MSH6*	c.2633T>C	rs2020912	p.(Val878Ala)	3	1
*MSH6*	c.3261dup	Not found	p.(Phe1088Leufs^*^5)	5	1
*MSH6*	c.3438+14A>T	rs2020911	p.(=)	1	19
*MSH6*	c.3438+14delinsTT	Not found	p.(=)	3	1
*MSH6*	c.3439-16C>T	rs192614006	p.(=)	2	1
*MSH6*	c.3514dup^3^	rs63751327	p.(Arg1172Lysfs^*^5)	5	1
*MSH6*	c.3699_3702dup^3^	Not found	p.(Leu1235Argfs^*^4)	4	1
*MSH6*	c.3804dup^3^	rs267608118	p.(Cys1269Metfs^*^6)	5	1
*MSH6*	c.3832_3845del^3^	Not found	p.(Pro1278Tyrfs^*^6)	4	1
*MSH6*	c.3848_3850dup^3^	Not found	p.(Ile1283dup)	3	1
*MSH6*	c.4001+12_4001+15del^3^	267608134	p.(=)	3	1
*MSH6*	c.4001+42_4001+45dup^3^	Not found	p.(=)	3	1
*MSH6*	c.^*^85T>A	rs2020906	p.(=)	2	2
*PMS2*	c.-154C>G	rs3735296	p.(=)	1	7
*PMS2*	c.52A>G^4^	rs63750123	p.(Ile18Val)	3	1
*PMS2*	c.59G>A	rs10254120	p.(Arg20Gln)	1	6
*PMS2*	c.251-72A>G	rs117831773	p.(=)	1	2
*PMS2*	c.288C>T^4^	rs12532895	p.(=)	1	6
*PMS2*	c.705+17A>G	rs62456182	p.(=)	1	20
*PMS2*	c.823C>T	Not found	p.(Gln275^*^)	4	1
*PMS2*	c.989-1G>T^4^,^5^	Not found	p.(=)	5	2
*PMS2*	c.1408C>T^4^	rs1805321	p.(Pro470Ser)	1	20
*PMS2*	c.1437C>G^4^	rs63750685	p.(His479Gln)	1	1
*PMS2*	c.1454C>A^4^	rs1805323	p.(Thr485Lys)	1	7
*PMS2*	c.1531A>G^4^	rs2228007	p.(Thr511Ala)	1	4
*PMS2*	c.1621G>A^4^	rs2228006	p.(Glu541Lys)	1	11
*PMS2*	c.1688G>T^4^	rs63750668	p.(Arg563Leu)	2	1
*PMS2*	c.1866G>A	rs1805324	p.(Met622Ile)	1	1
*PMS2*	c.1970del^3^	Not found	p.(Asn657Ilefs^*^8)	4	1
*PMS2*	c.2006+6G>A	rs111905775	p.(=)	1	7
*PMS2*	c.2007-4G>A	rs1805326	p.(=)	1	6
*PMS2*	c.2007-7C>T	rs55954143	p.(=)	1	6
*PMS2*	c.2156del^3^	Not found	p.(Gln719Argfs^*^6)	4	1

The columns shown in order are: Gene name, variant at DNA level, dbSNP rsID, variant at protein level, proposed class of variants, and number of samples the variants were detected in.

1 *PMS2* (NG_008466.1), *MSH6* (NG_007111.1), *MLH1* (NG_007109.1), and *MSH2* (NG 007110.1).

2 Variant classes are: 1 = neutral, 2 = likely neutral, 3 = uncertain, 4 = likely pathogenic, and 5 = pathogenic. Classification was done based on published literature, prediction tools, frequency (both publicly available and from our own diagnostic database) and conservation of nucleotides and amino acids.

3 Variants sequenced to test indel detection capabilities of the GS Junior platform.

4 *PMS2* variants confirmed by cDNA sequencing.

5 r[=]+[989_1015del].

### Variant calling, sensitivity and specificity

To assess insertion and deletion variant detection capabilities of the GS Junior platform, one run was specifically dedicated to this task. We sequenced 14 deletions, seven duplications, and two indel variants involving 1–14 bp and previously leftacterized with Sanger sequencing (footnoted 3 in Table 1). Two of these variants (c.680_683del in *MSH2* and c.2156del in *PMS2*) were located in or close to HP regions. All variants were successfully called in 40–56% of total reads. More comparable to a diagnostic sequencing setup, we also sequenced 16 samples containing 37 unique variants (35 substitutions and two indel variants) previously leftacterized by Sanger sequencing (run 1 and 2). Only variants with combined VF of at least 15% and present in both forward and reverse reads were considered. When only considering the amplicons previously Sanger sequenced, 315 variants met filter criteria. Of these, 146 were TP and 169 were FP variants. Of the FP variants, 154 originated from 26 different HP regions ranging from 5 to 8 bp. Cross-sample comparison and evaluation of signal distributions in HP regions reduced the number of FP to 18 and did not discard any of the TP variants. Of these, three calls were in two different HP regions and the remaining 15 FP calls came from two unique calls in *MSH2* (c.-243C>T) and *PMS2* (c.-175A>T). Both of these variants were most likely caused by nonrandom sequencing errors introduced by the SensiMix™ polymerase (Bioline), as they were not present in these fragments when amplified with AmpliTaq Gold 360 (Life Technologies). We are currently in the process of transferring singleplex amplification from SensiMixTM to AmpliTaq Gold 360. These two FP variants will therefore be eliminated in future runs. All 123 TP variants originating from 35 unique substitutions and two indel variants were detected. One of the indel variants, a deletion (c.1786_1788del in *MSH2*), was detected in ∼50% of both forward and reverse reads. The other, a duplication variant (c.3261dup in *MSH6*) located within a HP of eight repeats, was detected in 60% of the forward reads and 16% of the reverse reads, constituting 34% of total reads. Based on these results, the measured sensitivity (TP/(TP + FN)) of this validation set was 100%. However, the maximum sensitivity of this sample size (*n* = 60 different variants) is 95% (95 CI) because of an 5% (3/*n* = 3/60 = 0.05) probability of a FN event not being represented in this validation set (Mattocks et al. 2010). To calculate specificity, we used the fraction of TP among all positives (TP/(TP + FP)), also known as positive predictive value (PPV), instead of the standard specificity (TN/(TN + FP)). As all nucleotide positions coinciding with the reference sequence will be true negatives, the number of TN will be much larger than the number of FP. In this situation, the standard specificity will always be close to one, as the ratio will be dominated by TN, whereas the PPV will give a more informative value (Tompa et al. 2005; Zvelebil and Baum 2007). Hence, the specificity (PPV) after filtering was 46% (146/(146 + 169)). Cross-sample comparison and evaluation of signal distributions increased the specificity to 89%.

**Figure 3:**
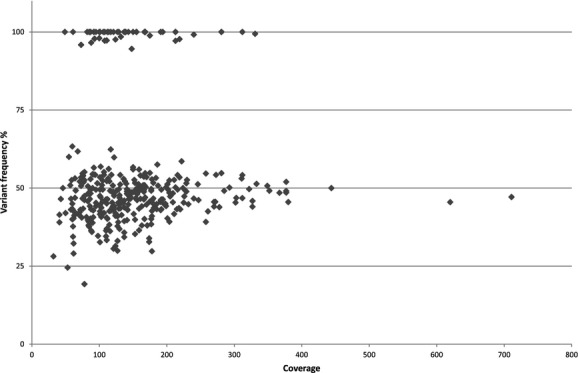
Variant frequency of all true variants identified in run 1–4 plotted against the coverage of their respective amplicons.

Theoretically, heterozygote and homozygote variants should be detected in 50% and 100% of the reads, respectively. In the four sequencing runs analyzed, the heterozygote detection range was from 19% to 63% of the reads (mean 46%). Homozygote variants were always found in >94% of the reads (mean 99%). Most of the TP variants with low detection frequency were located close to or in repetitive sequences. As an example, the variant with the lowest detection frequency (c.2006+6G>A in *PMS2*) was located between two short HP stretches (aaa*g*tttt). This is a commonly occurring polymorphism and was always called at relatively low frequencies indicating that sequence context in close proximity to the variant can affect base calling efficiency. To evaluate the effect coverage has on VF, we plotted all the TP variants identified in run 1–4 against the coverage obtained for their respective amplicons (Fig. 3). The VFs were more variable at lower coverage, especially below 100×. As the coverage increased the heterozygote VFs came closer to 50%. Because of no sampling effect, less variation was observed for homozygous variants, also at lower coverage.

### Variant annotation

An additional 16 samples (run 3 and 4) were sequenced to test the workflow. We utilized the possibility in AVA to filter variants according to a predefined status of the topical variant. All polymorphic (class 1) and FP variants identified in run 1 and 2 were defined as, accepted and rejected, respectively, and were used to filter variants identified in run 3 and 4. All other variants identified (new FPs and nonpolymorphic) were defined as putative by the software and required further investigation. In run 3, there were 34 different FP variants and 26 unique polymorphic variants recognized and filtered correctly as rejected and accepted, respectively, leaving 19 different variants defined as putative requiring further investigation. Of these, nine variants turned out to be FP calls in HP regions based on cross-sample comparison and evaluation of signal distributions, and one variant was a commonly occurring polymorphism. The remaining 10 variants were Sanger sequenced and confirmed nine nonpolymorphic (class 2–3) and one FP call in a HP region. The newly identified FP-and polymorphic variants were defined prior to run 4, in which only eight different variants were recognized as putative. Of these, seven variants remained putative after cross-sample comparison and evaluation of signal distributions. Sanger sequencing revealed that three variants were erroneous calls in HP regions and the remaining four were nonpolymorphic variants (class 2–3). Furthermore, 27 FP variants were recognized as rejected and 28 polymorphic variants as accepted. Indeed, the rejected variants were not totally disregarded; a visual inspection of variant frequencies and signal distributions (if necessary) was done to ensure that these calls were correctly assigned as FPs. All nonpolymorphic (class 2–5) variants identified in run 3 and 4 were confirmed by Sanger sequencing.

In total, we identified 72 distinct and true variants (Table 1) by MPS, of which 23 were likely pathogenic or pathogenic variants (class 4 or 5). We could not find reference single-nucleotide polymorphism ID number for 19 of the variants. However, 13 of these variants have previously been reported by Sjursen et al. (2010). The variants c.3699_3702dup (*MSH6*), c.3848_3850dup (*MSH6*), c.4001+42_400+45dup (*MSH6*), c.823C>T (*PMS2*), c.1970del (*PMS2*), and c.2156del (*PMS2*) have, to our knowledge, not previously been published. For *PMS2*, 20 distinct variants were identified and none of these were found to be pseudogene sequence-specific variants. To confirm that these variants truly originated from *PMS2,* we performed cDNA sequencing of seven samples representing most of the 14 *PMS2* variants located in exons or exon–intron boundaries. However, three of the variants (c.1970del, c.2156del and c.1866G>A) could not be analyzed because RNA samples could not be obtained from the patients carrying the specific variants. The variants c.59G>A and c.823C>T could not be confirmed due to poor RNA quality (RIN 4.10) of the sample used. All the remaining nine variants (footnoted 4 in Table 1) were confirmed by cDNA sequencing to originate from *PMS2* and not from any of the pseudogenes, indicating that our amplicon design is specific for amplification of *PMS2*. For the patients with disease-associated *PMS2* variants, no other disease causing variants were identified in any other MMR gene.

### Coverage results

**Figure 4:**
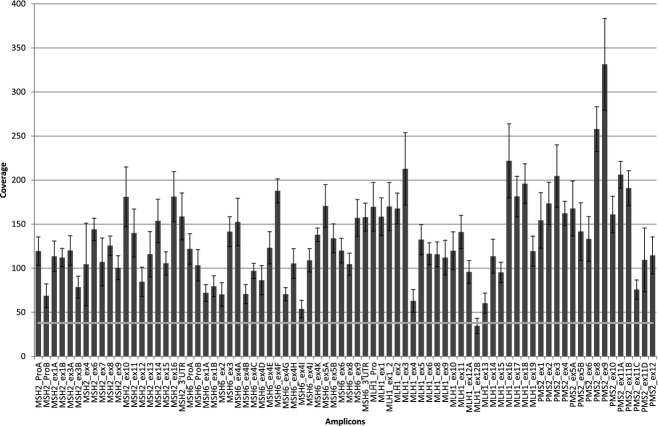
Distribution of coverage for each of the 78 amplicons for *MSH2*,*MSH6*,*MLH1*, and *PMS2* in our best performing run (run 4). Minimum coverage (38×) threshold is indicated with a light gray line. Seven amplicons all from a single-amplicon (MLH1_ex12B) was below the 38× threshold.

For MPS to be implemented in diagnostic settings, the method needs to be cost-effective. Uniform distribution of coverage across amplicons permits a larger sample size to be analyzed in a single-GS Junior run and thereby reduce sequencing costs. In our approach, the singleplex amplicons were pooled in different ratios to obtain uniform coverage distribution. Optimization was performed by adjusting the pool ratios of the amplicons prior each run, based on the coverage results obtained in the previous run. A summary of the coverage results for each run is shown in Table 2. We used the next generation molecular diagnostics calculator developed by De Leeneer et al. (2011a) to calculate the “spread correction factor” and number of samples that can be included in each sequencing run. An excellent explanation for how to calculate how many samples that can be included in a sequencing run (based on spread correction factor) is also given in another study by this group (De Leeneer et al. 2011b). In our best optimized run (run 4), we achieved a spread correction factor of 2.21 allowing us to pool and sequence 10 samples in a single run. Figure 4 shows the distribution of coverage of all the amplicons sequenced in run 4. For this run, seven amplicons had a coverage <38. All these were caused by a single amplicon (MLH1_ex12B), which require further optimization.

**Table 2:** Coverage results run 1–4.

	Run 1	Run 2	Run 3	Run 4
Samples	8	8	8	8
Amplicons	624	624	624	624
Passed reads	143,904	116,190	90,725	118,367
Mapped reads	120,796	87,873	68,973	82,598
Min/avg/max	0/194/791	13/141/873	0/111/326	24/132/420
Coverage SD	108	87	51	53
Variation coefficient	0.56	0.62	0.46	0.40
Spread corr. factor 90%/95%	2.23/2.85	2.07/2.47	1.94/2.35	1.84/2.21
Sample capacity^1^ F90/F95	10/8	11/9	12/10	13/10
Amplicons with no coverage	1	0	4	0
# amplicons <38	9	13	14	7

1 Calculated based on average mapped reads (90,060) for the four sequencing runs.

**Figure 5:**
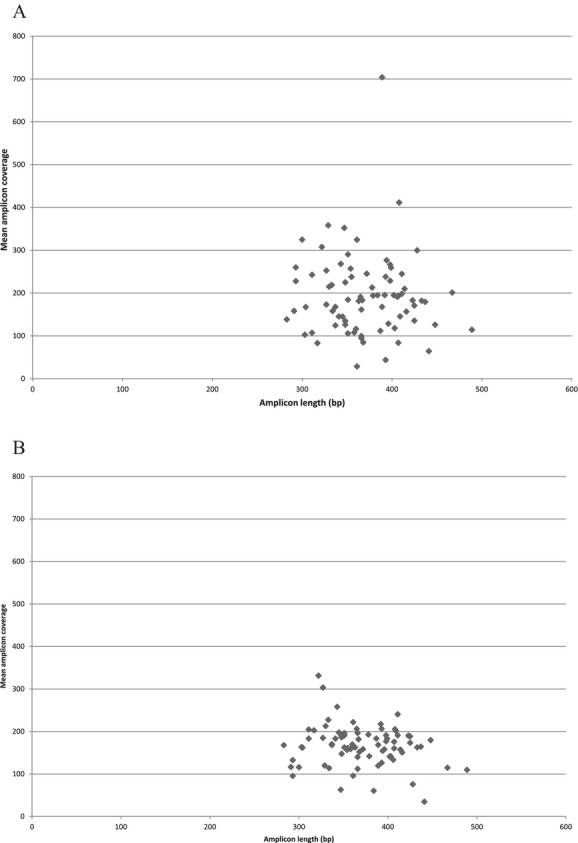
Correlation between amplicon length and the mean coverage for each amplicon in run 1 (A) and run 4 (B).

Pooling mixed-length amplicons in the second multiplex PCR and the emPCR should in theory cause a length bias where the shorter fragments are more efficiently amplified than the longer amplicons. If this was the case, long fragments should be pooled at higher ratios than short fragments. In our library, the amplicon lengths differ with up to 206 bp. However, we did not observe a relation between amplicon length and the ratio of which the amplicon was pooled (data not shown), indicating that other factors like sequence context may be of more importance for how efficiently an amplicon is amplified than the length (at least within the length range of the current study). Figure 5 shows a plot of the amplicon lengths against mean amplicon coverage in run 1 (before optimization) and run 4 (after optimization). The average coverage obtained for the different amplicons in run 4 is more focused around the mean value of 120 compared to run 1. This demonstrates that the approach of combining amplicons at different ratios based on their obtained coverage, efficiently counterbalance any effect length bias or sequence context has on amplification efficiency.

### Time and cost evaluation

We compared the consumable costs and time used from singleplex PCR to the final result between the MPS workflow and our previous Sanger sequencing workflow for eight samples. We evaluated the hands-on time to be similar for the two approaches, while consumable costs were reduced to approximately one-third. Sample turnaround time was also substantially reduced from 14 days to 6 days, mainly attributed to shorter run-time on the GS Junior platform and more efficient data analysis.

## Discussion

Increasing demand for genetic testing combined with the request of shorter turnaround times required a shift in sequencing methodology in our laboratory. Therefore, we evaluated if genetic testing using the GS Junior benchtop sequencer from Roche was suitable for implementation in a diagnostic laboratory in terms of sensitivity, specificity, distribution of coverage, hands-on time, costs, and sample turnaround time. We have developed and optimized a MPS workflow for analysis of the four MMR genes associated with LS that fulfilled all the above mentioned requirements. All true variants identified with Sanger sequencing were also detected by MPS.

An in-house amplicon-enrichment approach was found to be most compatible with diagnostic MPS of MMR genes compared to other enrichment strategies. Our laboratory has performed diagnostic analysis of MMR genes for many years, and only minor changes to our original setup was necessary to make it compatible with the new MPS workflow. Alternative to our approach is enrichment by long-range PCR, which has the advantage of amplifying target genes with only a few PCRs (Hernan et al. 2012). However, this approach amplifies large portions of intronic sequences that have waste sequencing capacity and thereby increase sequencing costs. Commercial solid-or liquid-phase DNA-capture methods enrich target sequences by hybridisation to oligonucleotides (Albert et al. 2007; Gnirke et al. 2009). Variable selection efficiency across the target regions and nonspecific capture of homologous sequences such as pseudogenes are major limitations for this enrichment procedure (reviewed in ten Bosch and Grody 2008; Gnirke et al. 2009). Use of DNA-capture methods in enrichment of MMR genes can therefore cause unreliable variant detection in *PMS2* due to the presence of multiple pseudogenes. A recent study reported advances in data analysis to filter out detected pseudogene variants (Chou et al. 2010). Although this holds great promise, it still needs improvements and further validation before implementation in diagnostics. Another drawback with capturing is that the setup is fixed. Prior to sequence analysis of the MMR genes, immunohistochemistry (IHC) analysis is often performed to detect lack of expression of MMR-proteins in tumor tissue to guide which gene to target. As opposed to capturing, with amplicon-based methods, genes can be included in the sequencing runs according to IHC implications. Multiplex amplification of MMR genes using the commercial kit HNPCC MASTR™ (Multiplicom, Niel, Belgium) is another amplicon enrichment option. However, this kit does not offer specific amplification of PMS2. According to their own specification notes, exons 4, 5, 11–15 are nonspecific. This may cause unreliable variant detection in *PMS2*. Compared to other studies, we chose quite loose filter settings (15% combined VF and present in both forward and reverse reads) to separate TP from FP variants. More stringent VF cutoff will reduce the number of FPs, but increase the risk of false negatives (FNs). As recommended by De Leeneer et al. (2011a), two recent studies, where the application was detection of hereditary disease causing variants, used a VF cutoff of 25% to detect a heterozygote variant (minimum coverage of 38% and 99.9% detection power) (De Leeneer et al. 2011b; Feliubadalo et al. 2012). Yet another study used a VF threshold of 35% (Jiang et al. 2012). In this study, VF thresholds of 25% and 35% would cause two FN and 15 FN, respectively. This clearly demonstrates that if thresholds are set too high to reduce amount of FP (increase specificity) it will cause FNs, which is unacceptable in a diagnostic setting where the sensitivity is more important than the specificity. Looser filter settings will cause poor specificity, but the only consequence is more FP variants to deal with, which may increase the workload of confirmatory Sanger sequencing. A major source of FP in our sequencing runs were incorrect base calling in HP regions. Different strategies for how to overcome this problem has been proposed like commercial HP assays that analyze coding HPs with capillary electrophoresis (Feliubadalo et al. 2012) or HRM analysis (De Leeneer et al. 2011b). We chose an alternative approach where we Sanger sequenced five amplicons with HPs >13 repeats that caused severe base calling problems. The remaining HPs were analyzed by MPS. Cross-sample comparison and evaluation of signal distributions of variants called in HP regions proved to be an efficient approach to identify TP variants and substantially reduced the number of FP variants requiring Sanger sequencing.

We developed this workflow to be used in a clinical diagnostic setting and our results suggest that the GS Junior system reliably detects substitutions and small insertion and deletion variants. However, heterozygote variant calling can be affected by sequence context, especially at lower coverage. As the coverage increases, the VFs come closer to the theoretically expected value of 50%. These aspects need to be taken into consideration when determining VF and minimum coverage thresholds. Although frameworks have been suggested (De Leeneer et al. 2011a), there are currently no common guidelines for analysis and interpretation of MPS data for reliable variant detection. Filter settings that effectively distinguish signal from noise probably need to be experimentally determined by each laboratory as they are likely to be dependent on enrichment technique, MPS technology, and software.

Reliable variant detection in *PMS2* is challenging due to the presence of multiple pseudogenes. Strong homology between pseudogenes and the *PMS2* sequence makes it difficult to design primers specific to *PMS2*. We designed primers utilizing the differences between *PMS2* and the pseudogenes and our results indicate that the primers are specific to the *PMS2* gene, as no pseudogene-specific sequence variants were detected. In addition, nine *PMS2* variants were analyzed by cDNA sequencing and confirmed to truly originate from *PMS2*. However, sequence exchange between the 3′ region of *PMS2* and the pseudogene *PMS2CL*, my lead to inclusion of *PMS2CL* sequences into *PMS2* or vice versa (Hayward et al. 2007). In cases where pathogenic or pseudogene-specific sequence variants are detected, results will be confirmed by cDNA analysis or long-range PCR utilizing exon 10 (not present in pseudogene) as *PMS2*-specific primer location. Similar strategies have successfully been used in previous studies (Hayward et al. 2007; Etzler et al. 2008; Vaughn et al. 2010).

Detection of large deletions or duplications (whole exons or multiexons) should in theory be possible by MPS using relative ratios of reads. However, previous studies (De Leeneer et al. 2011b; Feliubadalo et al. 2012) have evaluated this method to be unreliable, due to the three amplification steps prior sequencing. Pending methodology improvements, we will continue to analyze large deletions and duplications in MMR genes with multiplex ligation-dependent probe amplification (MLPA).

Coverage uniformity across all amplicons is important to fully exploit the capacity of a GS Junior run. We performed coverage optimization by adjusting the pooling ratios of amplicons pooled from the singleplex PCRs to multiplex PCRs. This turned out to be an easy and effective way to achieve coverage uniformity. Our best performing run achieved a spread correction factor that outperformed a previously developed in-house method optimized by adjusting primer concentrations (De Leeneer et al. 2011b).

Using a molecule per bead ratio of 0.5, we achieved from 90725 to 143904 reads per run, which is substantially higher than guaranteed by Roche (70,000 reads/run). However, we lost about 30% of reads per run, due to short reads that could not be mapped to the reference sequences. We have identified these short reads as primer dimers originating from amplification of the *PMS2* gene. Although we further optimized the *PMS2* PCRs, the short fragments could not be eliminated. Our current workflow is optimized for sequencing 10 samples in a single-GS Junior run. Removal of these short sequences can increase sample size even further and therefore alternative cleanup methods will be carried out to resolve this problem.

To conclude, we find MPS with the GS Junior system to be suited for routine clinical diagnostics offering reduced costs and accelerated sample turnaround, without compromising sensitivity. Still, we hope for future improvements of the method to overcome the challenges of incorrect base calling in HP regions. Our MPS workflow for variant detection in the four MMR genes is now well-implemented in the hospital laboratory, although we still rely on Sanger sequencing for some of the amplicons and to confirm nonpolymorphic variants.

## Supporting Information

Table A1 Composition of the difference amplicon pools.
